# The effect of intravenous thrombolysis on patients with successful thrombectomy depends on final reperfusion grade: A retrospective study

**DOI:** 10.1111/cns.14227

**Published:** 2023-04-18

**Authors:** Tao Tang, Di Li, Man‐Hong Zhao, Aline M. Thomas, Chuang Chuang, Tie‐Ping Fan, Shen Li

**Affiliations:** ^1^ Department of Neurology and Psychiatry, Beijing Shijitan Hospital Capital Medical University Beijing China; ^2^ Department of Neurointervention Dalian Municipal Central Hospital Affiliated with Dalian Medical University Dalian China; ^3^ The Russell H. Morgan Department of Radiology and Radiological Sciences the Johns Hopkins University School of Medicine Baltimore Maryland USA; ^4^ China Medical University Shenyang China; ^5^ Beijing Institute of Brain Disorders Capital Medical University Beijing China

**Keywords:** outcome, reperfusion grade, stroke, thrombectomy, thrombolysis

## Abstract

**Aims:**

Although intravenous thrombolysis (IVT) has not shown confirmative effects on the outcomes of patients receiving successful thrombectomy, it might influence the outcomes of a subset of these patients. This study aims to evaluate whether the effects of IVT depend on final reperfusion grade in patients with successful thrombectomy.

**Methods:**

This is a single‐center, retrospective analysis of patients with an acute anterior circulation large‐vessel occlusion and a successful thrombectomy between January 2020 and June 2022. Final reperfusion grade was evaluated by the modified Thrombolysis in Cerebral Infarction (mTICI) score, which was dichotomized into incomplete (mTICI 2b) and complete (mTICI 3) reperfusion. The primary outcome was functional independence (90‐day modified Rankin Scale score 0–2). Safety outcomes were 24‐h symptomatic intracranial hemorrhage and 90‐day all‐cause mortality. Multivariable logistic regression analyses were used to assess the interactions between IVT treatment and final reperfusion grade on outcomes.

**Results:**

When comparing all 167 patients enrolled in the study, IVT did not influence the extent of functional independence (adjusted OR: 1.38; 95% CI: 0.65–2.95; *p* = 0.397). The effect of IVT on functional independence depended on final reperfusion grade (*p* = 0.016). IVT benefited patients with incomplete reperfusion (adjusted OR: 3.70; 95% CI 1.21–11.30; *p* = 0.022), but not those with complete reperfusion (adjusted OR: 0.48, 95% CI: 0.14–1.59; *p* = 0.229). IVT was not associated with 24‐h symptomatic intracerebral hemorrhage (*p* = 0.190) or 90‐day all‐cause mortality (*p* = 0.545).

**Conclusions:**

The effect of IVT on functional independence depended on final reperfusion grade in patients with successful thrombectomy. IVT appeared to benefit patients with incomplete reperfusion, but not those with complete reperfusion. Because reperfusion grade cannot be determined prior to endovascular treatment, this study argues against withholding IVT in IVT‐eligible patients.

## Graphical abstract

This study showed that final reperfusion grade modified the effect of intravenous thrombolysis on functional independence in patients with acute anterior circulation large‐vessel occlusion and successful thrombectomy. Intravenous thrombolysis appeared to benefit patients with incomplete reperfusion, but not those with complete reperfusion.

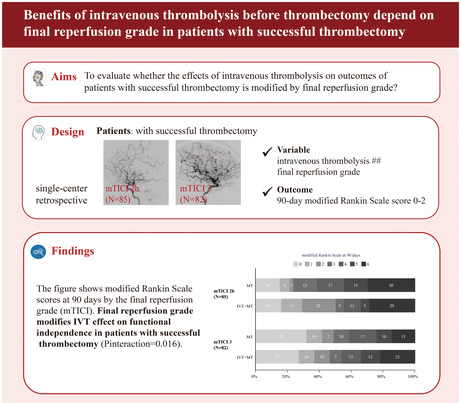

## 1 INTRODUCTION

With the steady advancement of mechanical thrombectomy (MT), the necessity of intravenous thrombolysis (IVT) before MT has been questioned,^1^ especially when the risk for hemorrhage is considered.^2^ A recent meta‐analysis of randomized clinical trials revealed comparable functional and safety outcomes in IVT‐eligible patients that received bridging therapy or direct MT.^3^ Therefore, personalized reperfusion strategies concerning IVT administration before MT might be a better option.^4^ Therefore, it is imperative to determine which candidate patients may benefit from IVT prior to MT.

Recent studies have evaluated the interactions between IVT and baseline variables on MT outcomes such as occlusion site,^5^ collateral status,^6^ Alberta Stroke Program Early CT Score (ASPECTS),^7^ in‐hospital treatment delay,^8^ and initial MT techniques.^9^ Meanwhile, final reperfusion grade, commonly evaluated by the modified Thrombolysis in Cerebral Infarction (mTICI) score,^10^ might be another potentially relevant factor. IVT was found to be associated with improved functional outcome amongst patients with unsuccessful MT (mTICI 0–2a),^11^ yet in a recent real‐world, observational study, this benefit was not observed in patients with anterior circulation large‐vessel occlusion following successful reperfusion (mTICI 2b‐3).^12^ Still, it is worth noting that the final reperfusion status varies substantially amongst patients with successful reperfusion.^13^ While a status of mTICI 3 indicates 100% reperfusion of the target territory, mTICI 2b indicates 50%–99% reperfusion.^10^ In addition, it is proposed that mTICI 2b is typically caused by procedure‐related distal vessel occlusions,^4^ which might respond to pre‐MT IVT. Thus, it is important to investigate whether the treatment effect of IVT depends on final reperfusion grade in patients with successful reperfusion.

## 2 METHODS

### 2.1 Study design and patient selection

Between January 2020 and June 2022, consecutive patients who underwent MT at Dalian Municipal Central Hospital for an acute ischemic stroke with large‐vessel occlusion were recruited into this retrospective study. Patients were included if they (1) had a proximal anterior circulation occlusion (intracranial internal carotid artery or middle cerebral artery (M1 or M2 segment) occlusions, or both), (2) were treated with MT within 6 hours from stroke onset, (3) were older than 18 years, (4) had a National Institutes of Health Stroke Scale (NIHSS) score ≥ 6, an ASPECTS ≥ 6, and a pre‐stroke modified Rankin Scale (mRS) ≤ 2, (5) had a successful reperfusion defined by a final mTICI score of 2b or 3, and (6) had a functional outcome assessment using mRS at 90 days. IVT with 0.9 mg/kg of alteplase was administered to eligible patients with written informed consent obtained according to current management guidelines.^14^ These patients were assigned into the incomplete reperfusion group (mTICI 2b) or the complete reperfusion group (mTICI 3) according to the final reperfusion grade, and further divided into IVT + MT and direct MT subgroups based on IVT treatment assignment.

The Dalian Municipal Central Hospital Ethics Committee approved the study (2019–004–11). Each patient gave written informed consent on admission for all diagnostic and therapeutic procedures and was informed that non‐personal information may be used for clinical investigations. All relevant information was obtained from the clinical database of the Dalian Municipal Central Hospital without re‐informing the patients because of the retrospective approach. Only anonymized data was used, and the patient privacy was not violated. The study was conducted according to the principles expressed in the Declaration of Helsinki.

### 2.2 Data collection

The following variables were collected: age, sex, medical history including main vascular risk factors and related treatments (hypertension, diabetes mellitus, previous ischemic stroke or transient ischemic attack (TIA), atrial fibrillation, antiplatelet or anticoagulation treatment, smoking status), pre‐stroke mRS, systolic blood pressure (SBP) and diastolic blood pressure (DBP) at admission, baseline NIHSS score and ASPECTS, administration of IVT, occlusion site, pre‐MT collateral status, anesthesia type, time from symptom onset to groin puncture, time from onset to reperfusion, device‐pass number, final reperfusion grade, and stroke etiology according to the Trial of Org 10,172 in Acute Stroke Treatment classification (TOAST).^15^ The pre‐stroke mRS was assessed by reception neurologists during medical history collection and during the physical examination. The pre‐MT collateral status was dichotomized into good (grade 3–4) and poor (grade 0–2) collaterals according to the American Society of Interventional and Therapeutic Neuroradiology/Society of Interventional Radiology collateral flow grading system.^16^ Imaging variables were assessed by two experienced neurointerventionalists blinded to patient information, with a consensus reading in the case of discrepancies.

### 2.3 Outcome evaluation

The primary outcome was functional independence (mRS ≤ 2) at 90 days after stroke, assessed by stroke neurologists during the clinical follow‐up visit (28/167) or via a standardized telephone interview with the patients or their caregivers (139/167). Safety outcomes were the incidence of 24‐h symptomatic intracranial hemorrhage (SICH) and 90‐day all‐cause mortality rate. SICH was defined as evidence of intracranial hemorrhage associated with an increase of 4 or more points on the NIHSS scores.^17^

### 2.4 Statistical analysis

The Shapiro–Wilk test was used to test data distribution. Categorical variables were expressed as frequencies and percentages. Continuous variables were expressed as mean (SD) or median [interquartile range (IQR)] in the case of non‐normal distribution. Baseline characteristics were compared using the Student *t* test/Mann–Whitney *U* test, or *χ* ^2^ test/Fisher's exact test, as appropriate, in complete and incomplete refusion groups.

We assessed whether the effects of pre‐MT IVT on primary and safety outcomes depended on final reperfusion grade (mTICI 2b versus mTICI 3) using multivariable binary logistic regression analysis. To select the variables included in the multivariable logistic regression, we first compared the baseline variables with outcomes using univariable analysis. Then, a backward stepwise multivariable binary logistic regression analysis was performed to identify variables that affected the outcomes. Variables with *p* < 0.10 in the univariate analysis were added to the multivariable analysis. *p* ≥ 0.10 of the likelihood ratio test was used as exclusion criteria from the final backward stepwise analysis. Finally, an interaction term (IVT × final reperfusion grade) was added into the final multivariable analysis to determine whether IVT's effects depended on final reperfusion grade. We reported unadjusted odds ratios (OR) for univariate analyses and adjusted OR for multivariable analyses with a 95% confidence interval (CI). All tests were 2‐tailed with a significance level of 0.05. All analyses were performed with the STATA software (version 17.0, StataMP, StataCorp).

## 3 RESULTS

### 3.1 Baseline characteristics

**FIGURE 1:**
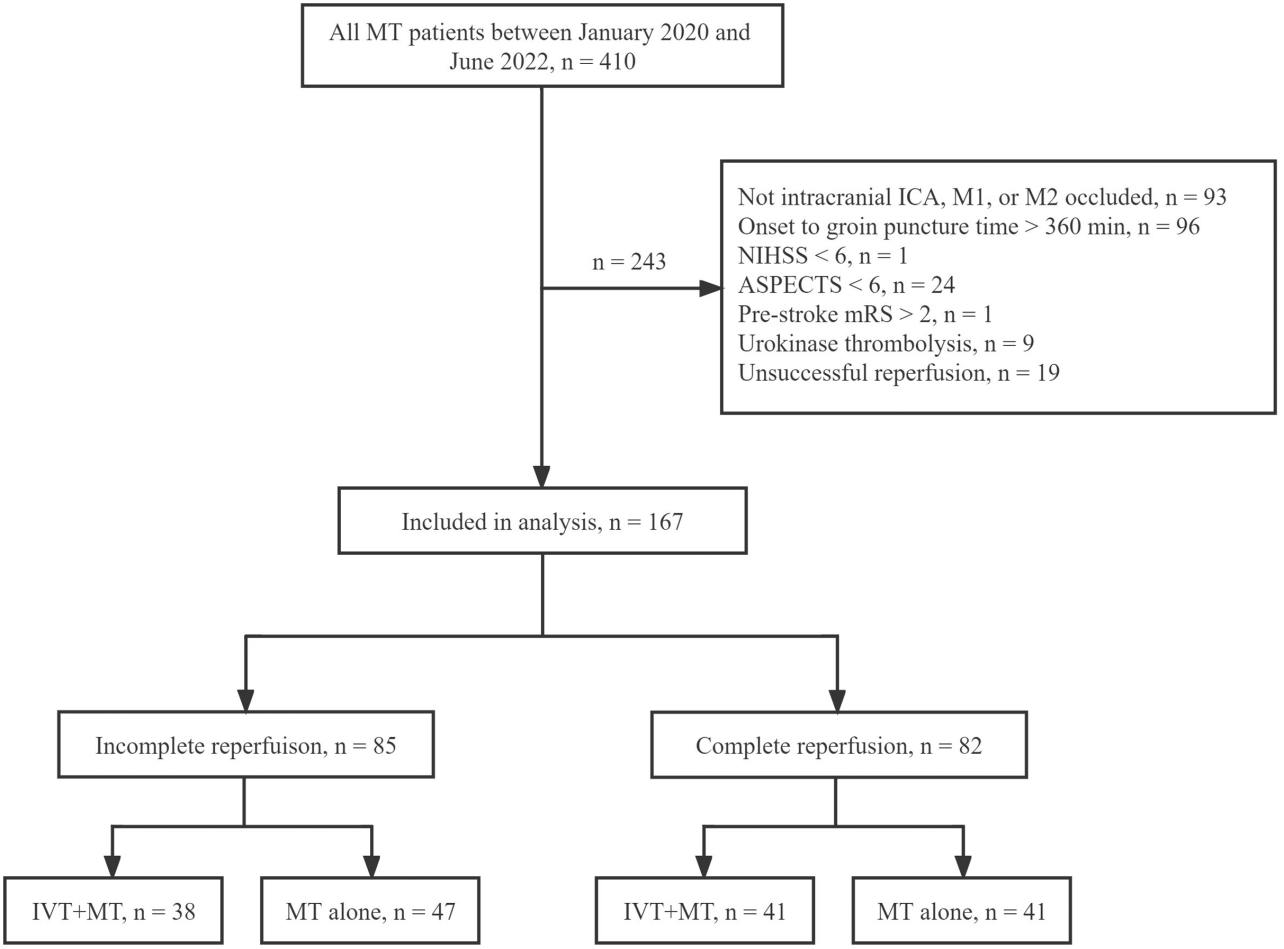
Flowchart illustrating the study inclusion/exclusion and grouping processes. ICA, internal carotid artery; MCA, middle cerebral artery.

A total of 167 patients were enrolled in the study. Eighty‐five patients had incomplete reperfusion, of which 38 underwent IVT + MT. Eighty‐two patients had complete reperfusion, of which 41 received IVT + MT (Figure 1). The baseline characteristics of the study cohort are shown in Table 1, which were balanced in both groups.

**TABLE 1:** Baseline characteristics stratified by both final reperfusion grade and IVT treatment assignment.

	Incomplete reperfusion (mTICI 2b, *n* = 85)			Complete reperfusion (mTICI 3, *n* = 82)		
	IVT + MT (*n* = 38)	Direct MT (*n* = 47)	*p* Value	IVT + MT (*n* = 41)	Direct MT (*n* = 41)	*p* Value
Age, median (IQR), years	70 [61–77]	68 [63–77]	0.951	69 [66–74]	73 [64–79]	0.220
Sex, female, *n* (%)	11 (28.9)	19 (40.4)	0.271	16 (39)	16 (39)	1.000
*Medical history, n (%)*						
Hypertension	20 (52.6)	23 (48.9)	0.735	22 (53.7)	20 (48.8)	0.659
Diabetes mellitus	6 (15.8)	11 (23.4)	0.383	16 (39)	11 (26.8)	0.240
Ischemic stroke/TIA	2 (5.3)	9 (19.1)	0.058	5 (12.2)	6 (14.6)	0.746
Atrial fibrillation	17 (44.7)	24 (51.1)	0.562	16 (39)	23 (56.1)	0.122
Antiplatelet treatment	1 (2.6)	4 (8.5)	0.252	5 (12.2)	2 (4.9)	0.236
Anticoagulation treatment	1 (2.6)	5 (10.6)	0.152	2 (4.9)	7 (17.1)	0.077
Smoking	17 (44.7)	17 (36.2)	0.423	13 (31.7)	15 (36.6)	0.641
*Pre‐stroke mRS, median (IQR)*			0.091			1.000
0	37 (97.4)	41 (87.2)		39 (95.1)	39 (95.1)	
1–2	1 (2.6)	6 (12.8)		2 (4.9)	2 (4.9)	
*Current stroke event*						
SBP, mean ± SD, mmHg	141.5 ± 23	144.3 ± 29.2	0.630	156.8 ± 26.6	151.3 ± 30.8	0.391
DBP, mean ± SD, mmHg	80.8 ± 12	80.8 ± 14.5	0.993	85.9 ± 15.8	82.9 ± 17.9	0.422
Baseline NIHSS score, median (IQR)	16 [14–20]	18 [15–25]	0.057	15 [12–20]	18 [13–23]	0.156
ASPECTS, median (IQR)	9 [7–10]	9 [8–10]	0.521	9 [8–10]	8 [7–10]	0.354
*Occlusion site, n (%)*			0.904			0.656
Intracranial ICA	14 (36.9)	18 (38.3)		18 (43.9)	19 (46.3)	
M1	23 (60.5)	27 (57.4)		15 (36.6)	17 (41.5)	
M2	1 (2.6)	2 (4.3)		8 (19.5)	5 (12.2)	
*Collateral status, n (%)*			0.515			0.641
Poor	25 (65.8)	34 (72.3)		26 (63.4)	28 (68.3)	
Good	13 (34.2)	13 (27.7)		15 (36.6)	13 (31.7)	
*Anesthesia*			0.389			0.477
General anesthesia	3 (7.9)	1 (2.1)		1 (2.5)	3 (7.3)	
Local anesthesia	19 (50.0)	22 (46.8)		24 (58.5)	20 (48.8)	
Conscious sedation	16 (42.1)	24 (51.1)		16 (39.0)	18 (43.9)	
Onset to groin puncture time, median (IQR), min	220 [170–278]	238 [160–280]	0.870	220 [168–274]	205 [155–275]	0.777
Onset to reperfusion time, median (IQR), min	295 [215–345]	291 [237–348]	0.884	281 [236–325]	267 [212–335]	0.882
Device‐pass number, median (IQR)	2 [1–3]	2 [1–3]	0.948	1 [1–2]	2 [1–2]	0.347
*Stroke etiology, n (%)*			0.655			0.348
Cardioembolism	23 (60.5)	27 (57.5)		24 (58.5)	24 (58.5)	
Large‐artery atherosclerosis	15 (39.5)	19 (40.4)		16 (39.0)	13 (31.7)	
Others	0 (0.0)	1 (2.1)		1 (2.5)	4 (9.8)	

Abbreviations: ICA, internal carotid artery; M1 and M2, the first and second segment of middle cerebral artery, respectively.

### 3.2 Primary outcome

**FIGURE 2:**
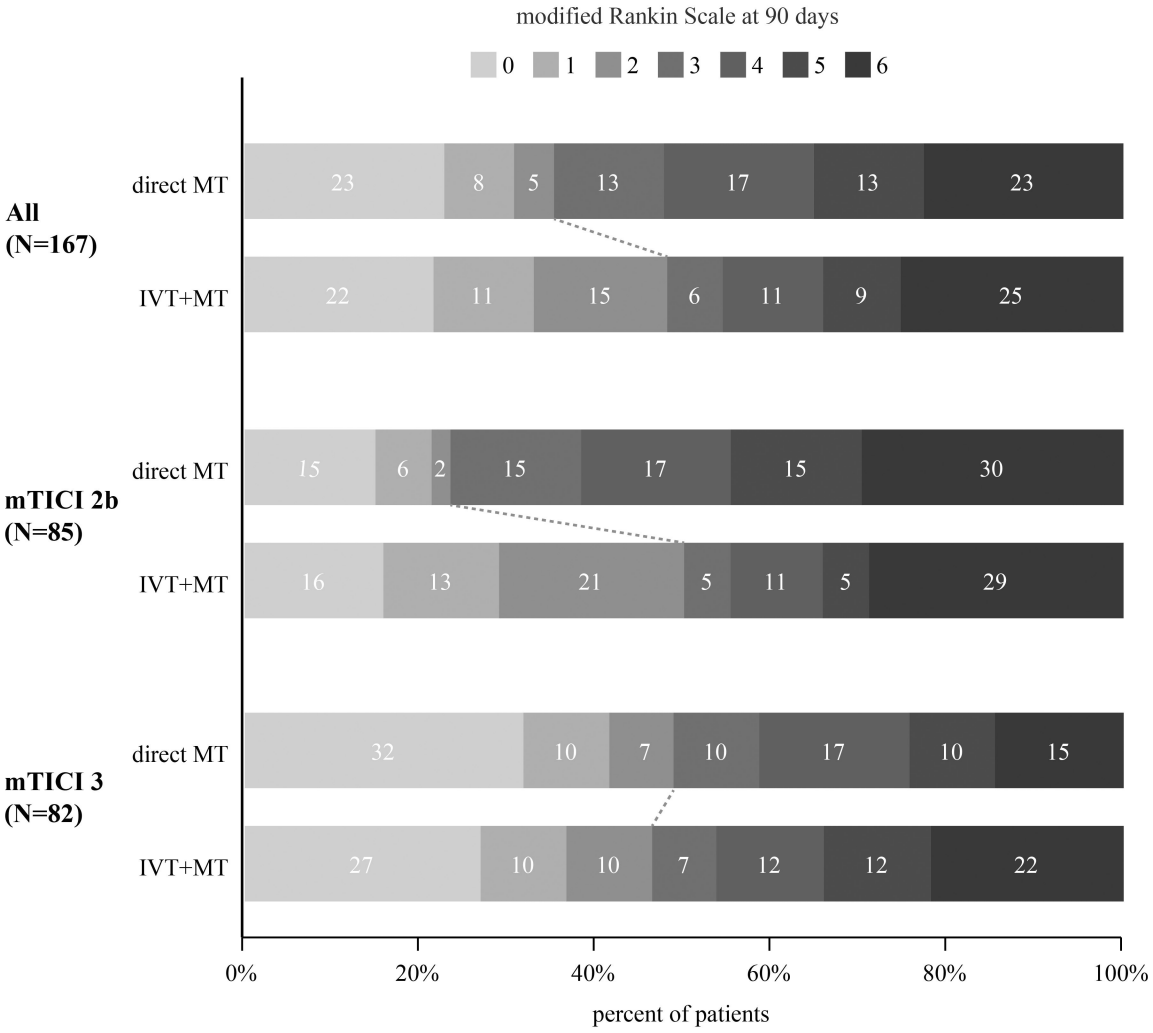
Distribution of mRS scores at 90 days post stroke in the whole patient cohort and by the final reperfusion grade. IVT was not associated with functional independence in the overall patient population. IVT appeared to benefit patients with incomplete reperfusion but not those with complete reperfusion.

When the whole cohort was compared, the IVT + MT and direct MT groups had similar functional independence (48.1% vs 35.2%; adjusted OR: 1.38; 95% CI: 0.65–2.95; *p* = 0.397; Table 2, Figure 2). There was significant interaction between IVT and final reperfusion grade on functional independence (*p* = 0.016; Table 2). In the incomplete reperfusion group, patients treated with IVT + MT achieved more functional independence compared to those treated with direct MT (50.0% vs 23.4%; adjusted OR: 3.70; 95% CI: 1.21–11.30; *p* = 0.022). In the complete reperfusion group, functional independence did not differ between patients treated with or without IVT (46.3% vs 48.8%; adjusted OR: 0.48; 95% CI: 0.14–1.59; *p* = 0.229; Table 2, Figure 2).

**TABLE 2:** Intravenous thrombolysis (IVT) on functional and safety outcomes.

Outcome, *n* (%)	IVT + MT	Direct MT	OR (95% CI)	*p* Value	aOR (95% CI)	*p* Value	*p* Value for interaction
*Functional independence*							
All patients	38/79 (48.1)	31/88 (35.2)	1.70 (0.92–3.17)	0.093	1.38 (0.65–2.95)	0.397^b^	
Patients with mTICI 2b	19/38 (50.0)	11/47 (23.4)	3.27 (1.29–8.27)	0.012^a^	3.70 (1.21–11.30)	0.022^a^ ^,^ ^b^	0.016^a^
Patients with mTICI 3	19/41 (46.3)	20/41 (48.8)	0.91 (0.38–2.16)	0.825	0.48 (0.14–1.59)	0.229^b^	
*SICH*							
All patients	4/79 (5.1)	11/88 (12.5)	0.37 (0.11–1.22)	0.104	0.44 (0.13–1.50)	0.190^c^	
Patients with mTICI 2b	2/38 (5.3)	7/47 (14.9)	0.61 (0.11–3.35)	0.572	0.32 (0.06–1.81)	0.200^c^	0.803
Patients with mTICI 3	2/41 (4.9)	4/41 (9.8)	0.88 (0.16–4.62)	0.875	0.51 (0.08–3.13)	0.471^c^	
*All‐cause mortality*							
All patients	20/79 (25.3)	20/88 (22.7)	1.15 (0.57–2.35)	0.696	1.26 (0.50–2.65)	0.545^d^	
Patients with mTICI 2b	11/38 (28.9)	14/47 (29.8)	0.61 (0.24–1.57)	0.306	1.02 (0.38–2.72)	0.963^d^	0.502
Patients with mTICI 3	9/41 (22.0)	6/41 (14.6)	1.34 (0.45–3.96)	0.595	1.78 (0.54–5.82)	0.340^d^	

^a^ *p* < 0.05.

^b^ Adjusted for age, diabetes, baseline NIHSS score, occlusion site, collateral status, device‐pass number, and final reperfusion grade.

^c^ Adjusted for baseline NIHSS score, device‐pass number, and final reperfusion grade.

^d^ Adjusted for smoking status, large‐artery atherosclerosis etiology, and final reperfusion grade.

### 3.3 Safety outcomes

IVT did not influence the incidence of SICH after successful MT, no matter reperfusion was incomplete (adjusted OR: 0.32; 95% CI: 0.06–1.81; *p* = 0.200) or complete (adjusted OR: 0.51; 95% CI: 0.08–3.13; *p* = 0.471). Similarly, IVT did not change the all‐cause mortality rate in patients with incomplete reperfusion (adjusted OR: 1.02; 95% CI: 0.38–2.72; *p* = 0.963) or complete reperfusion (adjusted OR: 1.78; 95% CI: 0.54–5.82; *p* = 0.340). Interactions between IVT and final reperfusion grade on SICH incidence or on all‐cause mortality rate were not observed (Table 2).

## 4 DISCUSSION

This study showed that the effect of IVT on functional independence depended on final reperfusion grade in patients with acute anterior circulation large‐vessel occlusion and successful thrombectomy. Amongst patients with incomplete reperfusion, IVT was significantly associated with functional independence without increasing SICH or mortality. In contrast, IVT did not have an effect on functional independence in patients with complete reperfusion.

Final reperfusion status is a key factor influencing clinical outcomes of patients that have a large‐vessel occlusion treated with MT.^4^ Thus, successful reperfusion is the goal of MT.^18^ Complete reperfusion increases the likelihood of functional independence at 90 days by 15%–20% over incomplete reperfusion.^13^ However, despite continuous technical improvement in MT to increase the rate of complete reperfusion,^18^ more than 40% of patients still have incomplete reperfusion.^19^ In patients with failed MT, IVT was beneficial for regaining functional independence.^11^ However, a recent study showed that in 1872 patients that had successful reperfusion after anterior circulation large‐vessel occlusion, IVT was not associated with functional independence.^12^ This finding was confirmed in this study. Furthermore, we also revealed that IVT could improve functional independence in patients with incomplete reperfusion, but not in those with complete reperfusion.

Incomplete reperfusion is often believed to be caused by procedural‐related distal vessel occlusions,^4^ which could induce progressive infarction after MT. IVT is theoretically one effective treatment to avoid this embolic event. It might dissolve residual distal thrombi after MT,^4^ act on downstream microvascular thrombi, and/or promote microvascular patency,^20^ which could eventually improve cerebral blood flow and prevent infarct expansion. Therefore, we hypothesize that pre‐MT IVT may promote a delayed reperfusion in patients with incomplete reperfusion after MT that improves the functional independence.^12^, ^21^ Although there is a debate on the necessity of IVT before MT,^1^ our study indicates that withholding IVT treatment before MT might be the most prudent choice for current clinical practice, as final reperfusion status cannot be determined before the procedure. However, we should bear in mind that MT treatment should not be delayed in order to administer IVT, as prompt treatment can significantly improve MT outcomes.^22^

As an alternative to IVT for improving blood flow after partial recanalization, adjunctive intra‐arterial thrombolysis was recently proposed as a rescue strategy to achieve complete reperfusion in patients with incomplete reperfusion.^4^ This notion is supported by a study showing that adjunctive, intra‐arterial administration of alteplase resulted in a better neurological outcome at 90 days in patients with large‐vessel occlusion and successful reperfusion following MT, without increasing the odds of SICH or mortality.^23^ Although reperfusion of injured brain tissue provides an impetus for the development of post thrombolysis hemorrhagic transformation,^24^ pre‐MT IVT may also help to resolve embolisms of distal vessels, improve perfusion, and reduce the final volume of injured brain tissue.^13^ These benefits may balance the potentially increased bleeding risk,^24^ and result in a net benefit for patients with incomplete reperfusion.

This study has several limitations. First, it is a single‐center, retrospective study with a relatively small sample size, which could inevitably cause selection bias. However, the results obtained are both pathophysiologically plausible and clinically relevant. The sharpness of the outcome might be partially explained by the low data heterogeneity benefited from a single center study design. As such, a prospective study specifically designed to confirm the results obtained in this study is warranted. Second, the stroke neurologists assessing outcomes in this study were not blinded to patient information. To mitigate this limitation, the final reperfusion grade was evaluated by experienced neurointerventionalists blinded to patient information. Third, without advanced neuroimaging results, we can only suggest a hypothesis rather than a more concrete explanation of our findings. Perioperative dynamic perfusion imaging in future research may further elucidate potential mechanisms, such as improving microvascular compromise in no‐reflow phenomenon.^25^

## 5 CONCLUSIONS

The treatment effect of IVT on functional outcome depended on final reperfusion grade in patients with successful thrombectomy. IVT appeared to benefit patients with incomplete reperfusion, but not those with complete reperfusion. Because reperfusion grade cannot be determined prior to endovascular treatment, this study argues against withholding IVT in IVT‐eligible patients.

## AUTHOR CONTRIBUTIONS

TT, DL, and M‐HZ contributed to the work equally and should be regarded as the first authors. TT, DL, and SL designed the study. DL, M‐HZ, CC, and T‐PF collected the data. TT and SL did data analysis. TT, DL, M‐HZ, AMT, and SL drafted the manuscript and revised it critically. All authors read and approved the final version of the manuscript.

## CONFLICT OF INTEREST STATEMENT

The authors declare no conflict of interest.

## Data Availability

The data that support the findings of this study are available from the corresponding author upon reasonable request.

## References

[1] Gauberti M , Martinez de Lizarrondo S , Vivien D . Thrombolytic strategies for ischemic stroke in the thrombectomy era. J Thromb Haemost. 2021;19(7):1618‐1628. 33834615 10.1111/jth.15336

[2] Dicpinigaitis AJ , Gandhi CD , Shah SP , et al. Endovascular thrombectomy with and without preceding intravenous thrombolysis for treatment of large vessel anterior circulation stroke: a cross‐sectional analysis of 50,000 patients. J Neurol Sci. 2022;434:120168. 35101765 10.1016/j.jns.2022.120168

[3] Trifan G , Biller J , Testai FD . Mechanical thrombectomy vs bridging therapy for anterior circulation large vessel occlusion stroke: systematic review and meta‐analysis. Neurology. 2022;98(13):e1361‐e1373. 35173017 10.1212/WNL.0000000000200029

[4] Derraz I . The end of tissue‐type plasminogen activator's reign? Stroke. 2022;53(8):2683‐2694. 35506385 10.1161/STROKEAHA.122.039287

[5] Zhou Y , Xing P , Li Z , et al. Effect of occlusion site on the safety and efficacy of intravenous Alteplase before endovascular Thrombectomy: a Prespecified subgroup analysis of DIRECT‐MT. Stroke. 2022;53(1):7‐16. 34915738 10.1161/STROKEAHA.121.035267

[6] Anadani M , Januel AC , Finitsis S , et al. Effect of intravenous thrombolysis before endovascular therapy on outcome according to collateral status: insight from the ETIS Registry. J Neurointerv Surg. 2022;15:14‐19. 35115393 10.1136/neurintsurg-2021-018170

[7] Broocks G , Heit JJ , Kuraitis GM , et al. Benefit of intravenous alteplase before thrombectomy depends on ASPECTS. Ann Neurol. 2022;92:588‐595. 35801346 10.1002/ana.26451

[8] Meinel TR , Kaesmacher J , Buetikofer L , et al. Time to treatment with bridging intravenous alteplase before endovascular treatment:subanalysis of the randomized controlled SWIFT‐DIRECT trial. J Neurointerv Surg. 2022. neurintsurg‐2022‐019207. doi:10.1136/jnis-2022-019207. Online ahead of print. PMC10715486 35902234

[9] Rinkel LA , Treurniet KM , Nieboer D , et al. Effect of intravenous alteplase treatment on first‐line stent retriever versus aspiration alone during endovascular treatment. Stroke. 2022;53(11):3278‐3288. doi:10.1161/STROKEAHA.121.038390 35876018

[10] Zaidat OO , Yoo AJ , Khatri P , et al. Recommendations on angiographic revascularization grading standards for acute ischemic stroke: a consensus statement. Stroke. 2013;44(9):2650‐2663. 23920012 10.1161/STROKEAHA.113.001972 PMC4160883

[11] Rozes C , Maier B , Gory B , et al. Influence of prior intravenous thrombolysis on outcome after failed mechanical thrombectomy: ETIS registry analysis. J Neurointerv Surg. 2022;14(7):688‐692. 34413246 10.1136/neurintsurg-2021-017867

[12] Douarinou M , Gory B , Consoli A , et al. Impact of strategy on clinical outcome in Large vessel occlusion stroke successfully reperfused: ETIS registry results. Stroke. 2022;53(1):e1‐e4. 34727741 10.1161/STROKEAHA.121.034422

[13] Kaesmacher J , Ospel JM , Meinel TR , et al. Thrombolysis in cerebral infarction 2b reperfusions: to treat or to stop? Stroke. 2020;51(11):3461‐3471. 32993461 10.1161/STROKEAHA.120.030157

[14] Powers WJ , Rabinstein AA , Ackerson T , et al. Guidelines for the early management of patients with acute ischemic stroke: 2019 update to the 2018 guidelines for the early management of acute ischemic stroke: a guideline for healthcare professionals from the American Heart Association/American Stroke Association. Stroke. 2019;50(12):e344‐e418. 31662037 10.1161/STR.0000000000000211

[15] Adams HP Jr , Bendixen BH , Kappelle LJ , et al. Classification of subtype of acute ischemic stroke. Definitions for use in a multicenter clinical trial. TOAST. Trial of org 10172 in acute stroke treatment. Stroke. 1993;24(1):35‐41. 7678184 10.1161/01.str.24.1.35

[16] Anadani M , Finitsis S , Clarencon F , et al. Collateral status reperfusion and outcomes after endovascular therapy: insight from the endovascular treatment in ischemic stroke (ETIS) registry. J Neurointerv Surg. 2022;14(6):551‐557. 34140288 10.1136/neurintsurg-2021-017553

[17] von Kummer R , Broderick JP , Campbell BC , et al. The Heidelberg bleeding classification: classification of bleeding events after ischemic stroke and reperfusion therapy. Stroke. 2015;46(10):2981‐2986. 26330447 10.1161/STROKEAHA.115.010049

[18] Yeo LLL , Jing M , Bhogal P , et al. Evidence‐based updates to thrombectomy: targets, new techniques, and devices. Front Neurol. 2021;12:712527. 34566856 10.3389/fneur.2021.712527 PMC8459011

[19] Hill MD , Goyal M , Menon BK , et al. Efficacy and safety of nerinetide for the treatment of acute ischaemic stroke (ESCAPE‐NA1): a multicentre, double‐blind, randomised controlled trial. Lancet. 2020;395(10227):878‐887. 32087818 10.1016/S0140-6736(20)30258-0

[20] Desilles JP , Loyau S , Syvannarath V , et al. Alteplase reduces downstream microvascular thrombosis and improves the benefit of large artery recanalization in stroke. Stroke. 2015;46(11):3241‐3248. 26443832 10.1161/STROKEAHA.115.010721

[21] Mujanovic A , Jungi N , Kurmann CC , et al. Importance of delayed reperfusions in patients with incomplete thrombectomy. Stroke. 2022;53:3350‐3358. doi:10.1161/STROKEAHA.122.040063 36205143 PMC9586830

[22] Asdaghi N , Wang K , Gardener H , et al. Impact of time to treatment on endovascular thrombectomy outcomes in the early versus late treatment time windows. Stroke. 2023;54(3):733‐742. 36848428 10.1161/STROKEAHA.122.040352 PMC9991076

[23] Renu A , Millan M , San Roman L , et al. Effect of intra‐arterial alteplase vs placebo following successful thrombectomy on functional outcomes in patients with large vessel occlusion acute ischemic stroke: the CHOICE randomized clinical trial. JAMA. 2022;327(9):826‐835. 35143603 10.1001/jama.2022.1645 PMC8832304

[24] Yaghi S , Willey JZ , Cucchiara B , et al. Treatment and outcome of hemorrhagic transformation after intravenous alteplase in acute ischemic stroke: a scientific statement for healthcare professionals from the American Heart Association/American Stroke Association. Stroke. 2017;48(12):e343‐e361. 29097489 10.1161/STR.0000000000000152

[25] Wang X , Wang X , Ma J , et al. Association between the time of day at stroke onset and functional outcome of acute ischemic stroke patients treated with endovascular therapy. J Cereb Blood Flow Metab. 2022;42(12):2191‐2200. 35791272 10.1177/0271678X221111852 PMC9670006

